# Does the Neuroprotective Role of Anandamide Display Diurnal Variations?

**DOI:** 10.3390/ijms141223341

**Published:** 2013-11-27

**Authors:** Marina Martinez-Vargas, Julio Morales-Gomez, Ruben Gonzalez-Rivera, Carla Hernandez-Enriquez, Adan Perez-Arredondo, Francisco Estrada-Rojo, Luz Navarro

**Affiliations:** Physiology Department, School of Medicine, Universidad Nacional Autonoma de Mexico, AP 70-250, Mexico D.F. 04510, Mexico; E-Mails: marvar_69@yahoo.com.mx (M.M.-V.); julio_macaco181@hotmail.com (J.M.-G.); rubengonzalezrivera@yahoo.com.mx (R.G.-R.); medicosita.06@gmail.com (C.H.-E.); aparredondo23@yahoo.com.mx (A.P.-A.); fesro@hotmail.com (F.E.-R.)

**Keywords:** cannabinoid receptor, anandamide, circadian rhythms, cerebral cortex, neuroprotection

## Abstract

The endocannabinoid system is a component of the neuroprotective mechanisms that an organism displays after traumatic brain injury (TBI). A diurnal variation in several components of this system has been reported. This variation may influence the recovery and survival rate after TBI. We have previously reported that the recovery and survival rate of rats is higher if TBI occurs at 1:00 than at 13:00. This could be explained by a diurnal variation of the endocannabinoid system. Here, we describe the effects of anandamide administration in rats prior to the induction of TBI at two different times of the day: 1:00 and 13:00. We found that anandamide reduced the neurological damage at both times. Nevertheless, its effects on bleeding, survival, food intake, and body weight were dependent on the time of TBI. In addition, we analyzed the diurnal variation of the expression of the cannabinoid receptors CB1R and CB2R in the cerebral cortex of both control rats and rats subjected to TBI. We found that CB1R protein was expressed more during the day, whereas its mRNA level was higher during the night. We did not find a diurnal variation for the CB2R. In addition, we also found that TBI increased CB1R and CB2R in the contralateral hemisphere and disrupted the CB1R diurnal cycle.

## 1. Introduction

Recent *in vitro* and *in vivo* data have suggested that the endocannabinoid system is a component of mammalian neuroprotective mechanisms that an organism displays after suffering an insult such as traumatic brain injury (TBI) [1–4]. TBI triggers pathological pathways that may potentially harm brain cells. These mechanisms cause excitotoxicity, the formation of free radicals, inflammation, and apoptosis. However, autoprotective mechanisms are also activated by brain injury. An increase has been reported in the production of heat-shock proteins, anti-inflammatory cytokines, endogenous antioxidants, and endocannabinoids. These mediators have damage-reducing properties, and they are considered endogenous efforts to counteract traumatic damage and improve neuronal repair [5]. The balance between the harming and protective mechanisms ultimately determines the fate of the injured brain [5].

Several physiological characteristics of an organism have well-known circadian rhythms. The heart rate, core body temperature, and cardiovascular and metabolic events are a few of them. Besides physiological rhythms, the body often appears rhythmically susceptible to pathological events as well. In humans, asthma attacks occur more frequently in the early morning hours [6], migraine onsets peak at midday [7], and the incidences of myocardial infarctions [8] and ischemic strokes peak at approximately 9:00 [9,10]. In rats, a circadian rhythm of sensitivity to an ischemic insult produced by reversible middle-cerebral-artery occlusion has been reported [11].

We have previously reported that recovery from TBI is better in rats if the TBI occurs during the dark phase of the diurnal cycle [12]. We have also reported that the expression of the CB1R protein has a diurnal variation with a maximum expression at 13:00 and a minimum expression at 1:00 in both rat pons [13] and hippocampus [14], whereas its expression remains unchanged in the striatum [14].

The levels of endocannabinoids in some brain areas and the expression of some of the enzymes that synthesize or degrade them depend on diurnal variations [15–17]. Moreover, the brain’s circadian clock, the suprachiasmatic nucleus, exhibits high levels of expression of CB1R [18,19]. The administration of endocannabinoids has been shown to attenuate the ability of the circadian clock to entrain to light zeitgebers [20].

Considering all of these data, our study aimed to determine whether the neuroprotective role of anandamide (AEA) differs with the time of the day and if it is associated with the diurnal variation of CB1R or CB2R in the rat cerebral cortex.

## 2. Results and Discussion

**Figure 1.**
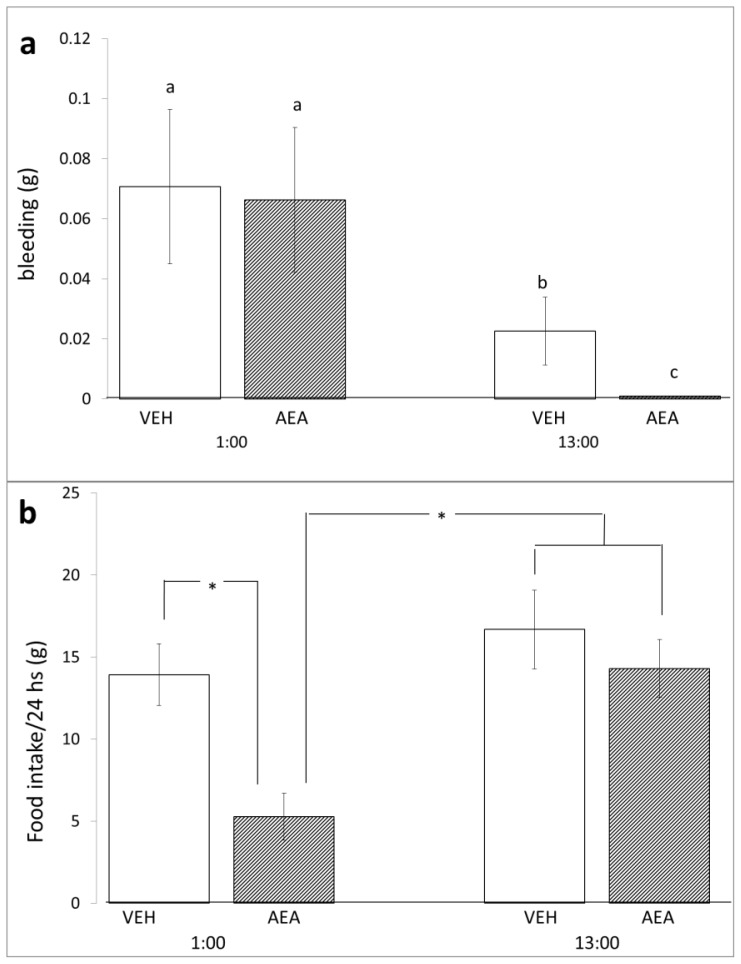

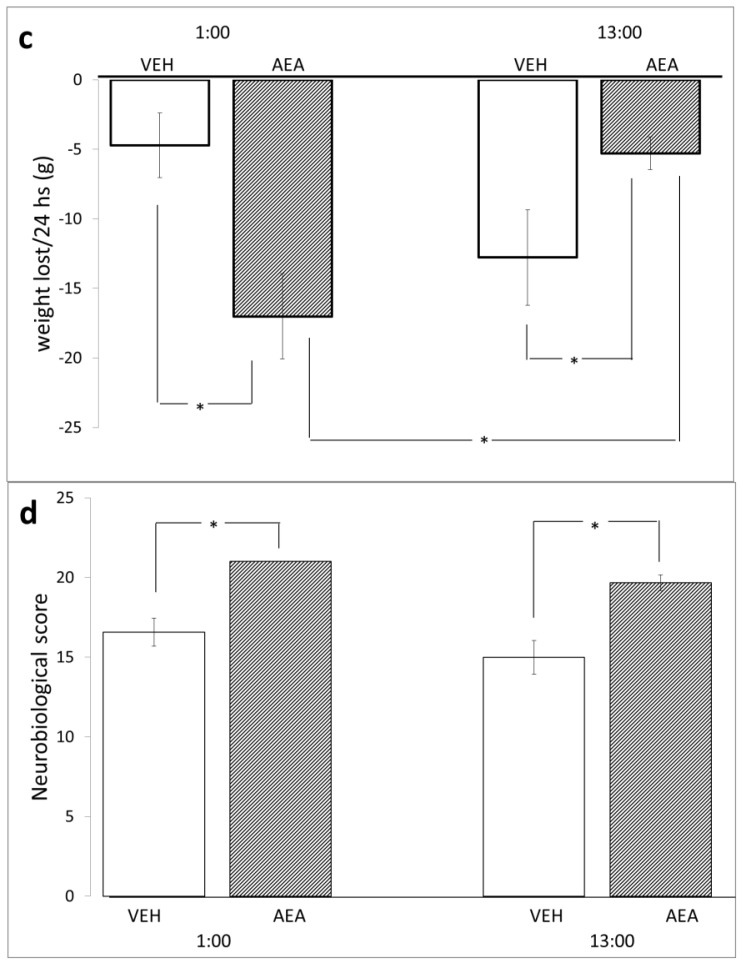
Effects of Traumatic Brain Injury (TBI) on bleeding, food intake, body weight differences, and neurobiological scores. The bars represent the means ± standard error of the mean (SEM) of (**a**) bleeding immediately after the TBI; (**b**) food intake; (**c**) body weight loss; and (**d**) neurobiological scores one day after TBI; (**a**) The bars with the same letter were not significantly different in a Kruskal–Wallis test and a *post-hoc* Mann–Whitney *U*-test; (**b**–**d**) * *p <* 0.05 in a one-way analysis of variance (ANOVA) and *post-hoc* Duncan test.

We observed that TBI performed at 13:00 produced less bleeding than that performed at 1:00, both in the vehicle group (0.023 ± 0.011 g *vs.* 0.071 ± 0.026 g; *Z* = −1.96, *p <* 0.05) and in the group treated with AEA (0 g *vs.* 0.066 ± 0.024 g; *Z* = −3.352, *p <* 0.001). These data were consistent with several reports indicating the existence of diurnal cycles that modulate platelet and endothelial functions as well as the concentration and activities of several proteins of the coagulation and fibrinolytic systems [21]. Significantly longer clotting times have been reported during the dark period in rats [22]. An AEA injection before the TBI significantly reduced hemorrhages when TBI was induced at 13:00 (0 g *vs.* 0.023 ± 0.011 g; *Z* = −2.591, *p <* 0.01) and had no effect on bleeding at 1:00 (0.066 ± 0.024 *vs.* 0.071 ± 0.026 g; *Z* = −0.530, *p* > 0.05) (see Figure 1a), which correlated with reports showing that AEA activates human [23] and rabbit [24] platelets. This effect was seen only when the TBI was performed at 13:00. AEA had no effects on bleeding when the TBI was induced at 1:00.

TBI impairs the physical state of the rat, and this manifests itself through decreased body weight and a decreased intake of food and water. This decrease is part of the metabolic response to trauma [25], and it has been used by other researchers to evaluate the neuroprotective effects of various substances in brain injury models [26]. We observed a significant decrease in food intake in the subjects injected with AEA before the induction of TBI at 1:00 than in the subjects treated with vehicle (5.3 ± 1.4 g *vs.* 13.9 ± 1.9 g); however, there was no effect at 13:00 (16.7 ± 2.4 g *vs.* 14.3 ± 1.8 g) (see Figure 1b). The group injected with AEA and subjected to TBI at 1:00 had the most noteworthy bodyweight decrease than the group treated with vehicle (−17.0 ± 3.1 g *vs.* −4.7 ± 2.3 g). However, the AEA-treated group subjected to TBI at 13:00 presented a lower weight decrease than that of the group treated with vehicle (−5.3 ± 1.2 g *vs.* −12.8 ± 3.4 g) (see Figure 1c). These findings were surprising because several reports have indicated that AEA causes an increase in food intake [27], although these reports have not been verified in rats subjected to TBI. In addition, we observed that AEA produced a lesser weight decrease than that of vehicle in rats subjected to TBI at 13:00.

The injection of AEA significantly reduced the neurological damage score, regardless of the time the TBI was performed (19.7 ± 0.5 *vs.* 15.0 ± 1.1; *Z* = −2.854; *p <* 0.004 at 13:00 and 21 ± 0 *vs.* 16.6 ± 0.9; *Z* = −2.438; *p <* 0.015 at 1:00) (see Figure 1d). This result was consistent with the fact that the endocannabinoid system is a component of the mammalian neuroprotective mechanisms that an organism displays after suffering an insult [4]. The neuroprotective effects of AEA have been well documented in several studies on *in vitro* and *in vivo* ischemia, neurotoxicity, and neuroinflammation [28–31]. These actions appear to be mediated through its binding to CB1R and CB2R cannabinoid receptors as well as to other receptors such as the vanilloid receptor [31].

**Figure 2.**
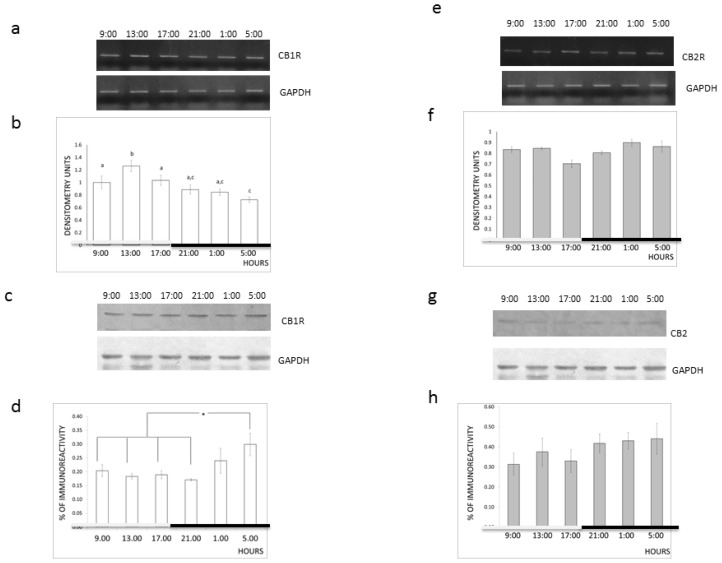
Effect of light-dark cycle on CB1R and CB2R expression. (**a**) Representative ethidium bromide-stained gel of polymerase chain reaction (PCR) fragments of CB1R and glyceraldehyde-3-phosphate dehydrogenase (GAPDH) in the rat cerebral cortex at different hours of the day; (**b**) Quantification of a negative ethidium bromide-stained gel by densitometry. The bars represent the mean ± SEM of the ratio of the densitometry units for CB1R and GAPDH (*n* = 4). The bars with the same letter were not significantly different in a one-way ANOVA and a Duncan *post-hoc*; (**c**) Representative picture of a western immunoblot of CB1R and GAPDH in the rat cerebral cortex at different hours of the day; (**d**) Quantification by densitometry of the western immunoblot bands. The bars represent the mean ± SEM of the ratio of the densitometry for CB1R and GAPDH (*n* = 4); * *p <* 0.05. One-way ANOVA and a Duncan *post-hoc* test; (**e**) Representative ethidium bromide-stained gel of PCR fragments of CB2R and GAPDH in the rat cerebral cortex at different hours of the day; (**f**) Quantification by densitometry of a negative ethidium bromide-stained gel. The bars represent the mean ± SEM of the ratio of the densitometry units for CB2R and GAPDH (*n* = 4); (**g**) Representative picture of a western immunoblot for CB2R and GAPDH in the rat cerebral cortex at different hours of the day; (**h**) Quantification by densitometry of the western immunoblot bands. The bars represent the mean ± SEM of the ratio of the densitometry obtained for CB2R and GAPDH (*n* = 4); *p <* 0.05. One-way ANOVA and a Duncan *post-hoc* test.

In addition, we examined the expression of CB1R and CB2R in the cerebral cortex of the rats killed at six different times of the day in order to analyze if the time-dependent effects of AEA correlated with the expression of CB1R and CB2R in the rat cerebral cortex. We observed diurnal variations in the expression of CB1R, both as protein (*F*_5, 18_ = 2.99; *p <* 0.039) and as mRNA (*F*_5, 18_ = 5.88; *p <* 0.002) (see Figure 2a–d) and no differences in the expression of CB2R, as protein (*F*_5, 18_ = 0.83; *p* > 0.05) or as mRNA (*F*_5, 18_ = 2.106; *p* > 0.05) (see Figure 2e–h).

We observed that the maximum values of CB1R mRNA levels occurred during the light phase (see Figure 2a,b), whereas the maximum values for the CB1R protein levels occurred during the dark period (see Figure 2c,d).

Previously, we have described diurnal variations in the levels of CB1R expression in the rat pons [13] and hippocampus [14]. Here, we observed a diurnal variation in the expression levels of CB1R in the rat cerebral cortex and no changes in the expression levels of CB2R. In contrast to the hippocampus and pons, the CB1R protein exhibited maximum levels of expression in the rat cerebral cortex during the dark period. Moreover, CB1R mRNA levels exhibited a diurnal variation with maximum values during the light phase. This shift of approximately 16 h between the maximum expression of the levels of CB1R mRNA and protein agreed with our previous findings of CB1R expression in the pons [13], where we measured a shift of about 16 h between the maximum levels of protein and mRNA. It was also in line with previously reported data for vasopressin (mRNA and peptide), which have indicated that peptide levels are high in the morning [32], whereas the mRNA levels are high in the afternoon [33].

This diurnal variation in the expression of CB1R is not a surprising finding because this type of phenomenon has been reported for other receptors. For example, a diurnal variation has been demonstrated in dopaminergic receptors [34–36].

Moreover, 2-arachidonoyl glycerol, which is considered the more abundant endocannabinoid [37], has a diurnal variation in the prefrontal cortex with a maximum level during the light phase [15], which correlates with the drop in CB1R expression levels in the cerebral cortex. It is difficult to claim that a downregulation mechanism controls the synthesis of CB1 receptors, although the literature supports an inverse relationship between the expression of CB1R protein and mRNA. Romero *et al.* [38] and Zhuang *et al.* [39] reported a decrease in the specific binding of the CB1 receptor in several tissues after an acute or long exposure to CB1R agonists. Remarkably, they also reported an increase in the expression of CB1R mRNA that correlates with the fact that cannabinoid agonists increase *CB1R* gene transcription in T cells [40].

**Figure 3.**
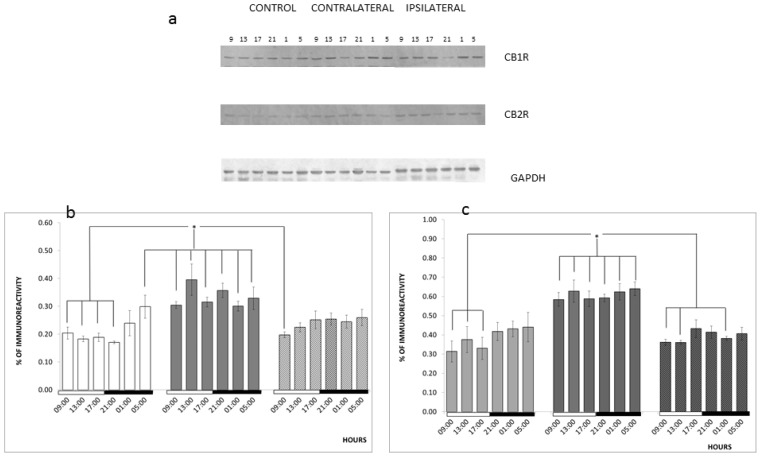
Effects of TBI on CB1R and CB2R expression. (**a**) Representative picture of a western immunoblot of CB1R, CB2R, and GAPDH in the rat cerebral cortex of rats subjected to TBI at different hours of the day; (**b**) Quantification by densitometry of the western immunoblot bands for CB1R. The bars represent mean ± SEM of the ratio of the densitometry for CB1R and GAPDH (*n* = 3); (**c**) Quantification by densitometry of the western immunoblot bands for CB2R. The bars represent mean ± SEM of the ratio of the densitometry for CB2R and GAPDH (*n* = 3); * *p <* 0.05. Two-way ANOVA and a Duncan *post-hoc* test.

We also analyzed the effects of TBI, induced at different times, on the expression of CB1R and CB2R. We found that TBI did not modify the expression levels of CB1R protein or CB2R protein in the ipsilateral hemisphere. Nevertheless, we found a significative increase in the CB1R protein levels (*F*_2, 54_ = 30.689, *p* < 0.001) and CB2R protein levels (*F*_2, 54_ = 49.519, *p* < 0.001) in the contralateral hemisphere, and; the diurnal variation of CB1R was abolished (*F*_5, 54_ = 1.528, *p* > 0.05; see Figure 3). This differential expression between the contralateral *versus* ipsilateral hemisphere has been recently documented. With a controlled unilateral cortical impact model, White *et al.* [41] reported that TBI is associated with a powerful proinflammatory response in ipsilateral brain tissues and a gene expression pattern in the contralateral side that suggests a remote anti-inflammatory response.

The changes in the levels of expression of these receptors in TBI models have not been analyzed before. However, in spinal cord injury, it has been reported that the expression of CB1R is constitutively expressed by neurons and oligodendrocytes and induced in reactive astrocytes, while CB2R is strongly upregulated after lesions and mostly expressed by immune infiltrates and astrocytes [42]. Furthermore, several authors have reported that ischemia induces changes in the levels of expression of CB1R and CB2R. For example, Jin *et al.* [43] reported that middle cerebral artery occlusion for 20 min causes an increase in the expression of CB1R in the area adjacent to the artery starting from 2 h after ischemia up to 72 h. However, Schomacher *et al.* [44] demonstrated that shorter periods of ischemia (2.5 min) reduce the expression of CB1R in the hippocampus of gerbils. Zhang *et al.* [45] demonstrated that middle cerebral artery occlusion in mice for 60 min causes an increase in the levels of CB1R mRNA expression in the first 6 h of reperfusion, which reached the base level after 24 h, whereas the levels of CB2R mRNA exhibit a decrease in the early hours of reperfusion and an increase at 24 h.

In addition, we showed that TBI disrupts the diurnal variation in the expression of the CB1 receptor in the brain cortex. Several authors have reported that TBI causes circadian deregulations, including altered homeostatic mechanisms such as the regulation of blood pressure, heart rate, body temperature [46], hormonal cycles [47], and the sleep-wake cycle in patients [48,49]. Recently, it has been shown that the expression of circadian genes is disrupted in the suprachiasmatic nucleus and hippocampus of rats that are subjected to TBI [50].

We analyzed the effects of an intracerebroventricular injection of AEA on survival and recovery before TBI was induced at two different times of day: at 13:00 (in the light) or at 1:00 (in the dark). We measured an apparent increase in the survival of rats injected with AEA before TBI at 13:00 (10% *vs.* 0%) and no differences at 1:00 (0% *vs.* 0%).

All of these results corroborated our previous report [12], which showed that the hour at which a subject experiences a TBI affects its life expectancy and recovery. Furthermore, in rats with no AEA administration, the recovery after a TBI was better when it was induced at night, a time when CB1R expression levels in the cerebral cortex were at its maximum level. Nevertheless, AEA administration appeared to be more effective when TBI was induced during the day. These data correlated with reports in the literature that suggest that the endocannabinoid system has the ability to modulate [20] or even invert the circadian rhythm [51]. We have reported that the intrahippocampal administration of AEA can switch the strategy used by rats to solve a maze during the light and dark phases of the photoperiod [14].

Furthermore, AEA administration modified the expression of CB1R and/or CB2R. According to what has been described by several authors, the chronic or semi-chronic administration (5–15 days) of a CB1R agonist [38,52,53] or FAAH inhibitors [54] decreases specific binding and induces endocytosis without changing or increasing the mRNA levels of CB1R [38,39,55], although there are exceptions depending on the model used. However, we did not examine this issue experimentally. There are only few studies, like ours, on acute administration of AEA. However, it has been reported that low doses of AEA or Δ^9^-THC induce an increase in the specific binding for CB1R [56] and that high doses of Δ^9^-THC induce a decrease in specific binding, particularly in the cerebral cortex [38,39], and in mRNA in the hippocampus, striatum, and cerebellum [39]. There are few reports on CB2; one study has described a marked increase in CB1 mRNA and no observed changes in CB2R in T cells incubated with Δ^9^-THC for 48 h [40].

One interpretation of our data is that intracerebroventricular AEA administration might decrease CB1R density without altering the CB2R and, thus, there would be an increased ratio of CB2R/CB1R, which is associated with improved neuroprotection. Zhang [45] used cerebral ischemic/reperfusion injury and Heller [57] used a model of spinal cord injury, and they reported that the combination of selective inhibition of CB1R and activation of CB2R resulted in the greatest neuroprotection.

## 3. Experimental Section

### 3.1. Subjects

Male Wistar rats (250–300 g) were maintained under a controlled dark–light cycle (12 h:12 h, lights on at 8:00) with food and water *ad libitum*. All animal experiments were performed according to institutional guidelines and were approved by the local ethics committee. Forty-two rats were used for drug administration and TBI experiments, and 30 were used for protein and mRNA determinations.

### 3.2. Intracerebroventricular Administration of Drugs and TBI

A stainless-steel cannula (23 gauge) was stereotactically implanted into the lateral ventricle according to the methods of the Paxinos and Watson Atlas [58] (*p* = 0.8, *L* = 1.5, *V* = −3.8). The entire procedure was performed while the animals were anesthetized with a mixture of ketamine (66 mg/kg), xylazine (0.26 mg/kg), and acepromazine (1.3 mg/kg). After eight days of recovery, the rats were housed individually, a measured amount of food was delivered, and their weight was recorded. After 24 h of housing, the rats were anesthetized with 6% chloral hydrate (400 mg/kg). Fifteen minutes before inducing TBI, AEA (Sigma-Aldrich Co. LLC, St. Louis, MO, USA; 1.25 μg/4 μL) or saline-5% EtOH (4 μL) was intracerebroventricularly injected (1 μL/min). We have used these doses and the type of administration previously [14,59]. A moderate head injury was produced at 1:00 or 13:00 by dropping a weight (90 g from 50 cm height) onto the intact skull at *p* = 4. This model is known as a closed head injury. A moderate-head injury was defined as an injury resulting in a mortality rate of less than 20%. We chose these hours because we had previously found a statistically significant difference in the recovery of rats if the TBI was induced at 1:00 compared to the one induced at 13:00 [12].

After causing the TBI, the following characteristics were evaluated: bleeding, neurological damage and mortality.

### 3.3. Bleeding

We evaluated the external hemorrhage produced by the TBI and compared the effects of AEA *versus* control by weighing the blood drained after producing the TBI. In brief, 15 min after the TBI, the blood was drained and collected by pipette and then put into microtubes and weighed as previously described [12].

### 3.4. Neurological Damage

We used a 21-point behavioral–neurological scale that has been described by Hunter *et al.* [60] to evaluate neurological damage 24 h after the TBI. We evaluated paw placement (4 points), righting reflex (1 point), horizontal bar equilibrium (3 points), slanting platform (3 points), rotation (2 points), visual fore-paw reaching (2 points), contralateral reflex (2 points), motility (2 points), and general condition (2 points). The maximum score (minimum damage) was 21.

Eight days after the TBI, the rats were anesthetized with sodium pentobarbital (100 mg/kg, intraperitoneal), perfused with 4% paraformaldehyde, and the brains were removed, frozen, and sectioned (thickness: 30 μm) on a cryostat. The brain sections were collected serially from bregma −0.92 to −5.8 at 300-μm intervals from the injured area and stained with cresyl violet to verify the position of the cannula. Only animals with the cannula at the lateral ventricle were included in the data analysis.

### 3.5. Diurnal Variations

Control rats were deeply anesthetized with sodium pentobarbital (100 mg/kg, intraperitoneal) and killed by decapitation at different times (9:00, 13:00, 17:00, 21:00, 1:00, or 5:00), with four rats at each time, to analyze the levels of CB1R and CB2R protein and mRNA. From each rat, the cerebral cortex was extracted and divided into two portions, and one portion was immediately homogenized with phosphate-buffered saline (PBS) and protease inhibitors to analyze the proteins. The other portion was homogenized with TRIzol to analyze the mRNA. We previously used these times to analyze the diurnal variations of the CB1R [13,14].

In addition, we used 18 more rats to analyze the effects of TBI on the diurnal variation of the levels of expression of CB1 and CB2. Rats, which were previously anesthetized with 6% chloral hydrate (400 mg/kg), were subjected to TBI at different times (9:00, 13:00, 17:00, 21:00, 1:00, or 5:00) with three rats at each time. After 24 h, the rats were deeply anesthetized with sodium pentobarbital (100 mg/kg intraperitoneal), killed by decapitation, and the cerebral cortex was dissected and stored at −70 °C until analysis.

### 3.6. Western Blotting

To assess the levels of CB1R or CB2R protein expression, homogenized tissues were centrifuged at 600 × *g* at 4 °C for 10 min. The supernatant was centrifuged at 39,000 × *g* at 4 °C for 15 min. A 12% analytical sodium dodecyl sulfate-polyacrylamide gel electrophoresis was performed as described elsewhere [61]. Briefly, the resuspended precipitates of tissue homogenates (15-μg protein) were mixed 1:1 with Laemmli buffer and heated (95 °C, 5 min) before loading onto a 0.75-mm thick gel. The samples were electrophoresed (150 V, 2 h), and the gels were then transferred onto a nitrocellulose membrane (GE Healthcare Life Sciences, Buckinghamshire, UK) and subjected to 100 V for 1 h at 4 °C. The nitrocellulose membrane was stained with Ponceau S, and each lane was cut in two pieces: one was used to analyze the levels of CB1R or CB2R, and the other was used to analyze the levels of GAPDH. Subsequently, the membrane was washed and incubated with 3% phosphate-buffered saline (PBS)-Tween, 10% nonfat dry milk, and 2% goat normal serum for 30 min at room temperature, which was followed by incubation with anti-CB1R (1:1000; Cayman Chemical Company, Ann Arbor, MI, USA), anti-CB2 (1:500; Santa Cruz Biotechnology, Inc., Santa Cruz, CA, USA), or anti-GAPDH CB2 (1:2000; Santa Cruz Biotechnology, Inc., Santa Cruz, CA, USA) overnight at 4 °C. The blot was washed with PBS-Tween (3 times × 5 min), incubated for 1 h at room temperature with goat anti-rabbit IgG horseradish peroxidase conjugate (1:2000), and developed with diaminobenzidine (0.5 mg/mL in PBS plus 0.3 μL/mL 30% H_2_O_2_). The density of the bands was analyzed using the Quantity One software (Bio-Rad Laboratories, Inc., Hercules, CA, USA).

### 3.7. RNA Extraction

To detect the changes in the expression of the CB1R and CB2R mRNA, total RNA was extracted using TRIzol according to the recommendations of the manufacturer (Life Technologies Corporation, Rockville, MD, USA). In brief, 100 mg of tissue was homogenized in 1 mL of TRIzol, and 200 μL of chloroform was added and mixed in a vortex mixer. Two phases were obtained by centrifugation at 12,000 × *g* for 10 min. The aqueous phase was recovered, and 0.5 mL of isopropyl alcohol was added. Total RNA was obtained by centrifugation at 12,000 × *g* for 10 min. Its integrity was confirmed by running an aliquot on a 1% agarose gel.

### 3.8. Reverse Transcriptase (RT)-PCR

RNA (2 μg) was reverse transcribed using the One Step System (Life Technologies Corporation, Rockville, MD, USA) and the recommendations of the manufacturer, with the addition of a step of DNAase treatment. Briefly, 2 μg of total RNA was incubated with DNAase (RNAase free), 1 U in 10 μL of the appropriate buffer solution, for 15 min at room temperature, and 1 μL of 25 mM ethylenediaminetetraacetic acid was added. The mixture was heated at 65 °C for 10 min to stop the reaction. This mixture was used for the RT reaction after adding 25 μL of buffer 2× (0.4 mM of each dNTP and 2.4 mM MgSO_4_), 1 μL of RT-*Thermus aquaticus* polymerase mixture, MgCl_2_, CB1R antisense primer (5′-ATGCTGTTGTCTAGAGGCTG-3′ (10 μM, 1 μL)) or CB2R antisense primer (5′-AGAACAGGGACTAGGACAAC-3′ (10 μM, 1 μL)), and water to adjust the volume to 50 μL. The RT was performed at 42 °C for 30 min and stopped by heating for 5 min at 94 °C. The PCR reactions used the following primers: CB1R sense primer: 5′-CATCATCATCCACACGTCAG-3′; CB1R antisense primer: 5′-ATGCTGTTGTCTAGAGGCTG-3′; or CB2R sense primer: 5′-GGAGTACATGATCTTGAGTGAT-3′; CB2R antisense primer: 5′-AGAACAGGGACTAGGACAAC-3′; 33 cycles, with denaturing for 45 s at 94 °C, annealing for 45 s at 53 °C, and elongation for 1 min at 72 °C. We also amplified the GAPDH cDNA to assess the RNA quality. We used the following primers: 5′-TCCCTCAAGATTGTCAGCAA-3′ and 5′-AATGTATCCGTTGTGGATCT-3′. These assay conditions were previously validated to assess that the amplifications fall on the upward logarithmic portion of the amplification plot. In all of the assays, RNA samples without RT were run to exclude DNA contamination.

### 3.9. Statistical Analyses

For the survival rate, the data were reported as a percentage, with significant differences determined with a chi-square test. For the other variables, the results were reported as mean ± SEM. Significant differences were determined using Kruskal–Wallis test and Mann–Whitney *U*-test for hemorrhage and the neurological score and one-way ANOVA and a Duncan post-hoc test for the other variables. *p* values less than 0.05 were considered statistically significant. Two-way ANOVA was used to compare the effects of TBI on the expression of CB1R and CB2R.

## 4. Conclusions

Our data showed that the neuroprotective effects of AEA administration had a diurnal variation and that the levels of expression of the CB1R in the rat cerebral cortex varied with the time of day. These effects should be taken into account when considering the potential clinical use of cannabinoids for the treatment of brain trauma.
